# Rate of urinary tract infection after urodynamic study in pelvic floor clinic

**DOI:** 10.22088/cjim.11.1.100

**Published:** 2020

**Authors:** Zinat Ghanbari, Fedyeh Haghollahi, Tahere Eftekhr, Tahere Froghifar, Mamak Shariat, Maryam Hajihashemy, Mohsen Ayati

**Affiliations:** 1 1.Department of Obstetrics and Gynecology, Vali-Asr Reproductive Health Research Center, Tehran University of Medical Sciences, Tehran, Iran; 2 Valiasr Reproductive Health Research Center, Tehran University of Medical Sciences, Tehran, Iran; 3 Baharloo Hospital, Depatment of Pelvic Floor (Urogynecology), Tehran University of Medical Sciences, Tehran, Iran; 4 Maternal, Fetal and Neonatal Research Center, Tehran University of Medical Sciences, Tehran, Iran; 5 Department of Obstetrics & Gynecology, Isfahan University of Medical Sciences, Isfahan, Iran; 6 Department of Uro-Oncology Research Center, Tehran University of Medical Sciences, Tehran, Iran

**Keywords:** Urinary tract infection, Pelvic floor, Urodynamic study

## Abstract

**Background:**

One of the complications of urodynamic study is urinary tract infection. The aim of this study was to determine the rate of urinary tract infection (UTI) after UDS in patients referred to the pelvic floor clinic with regard to the specific conditions of these patients, such as presence of pelvic organ prolapse and high post voiding residual volume (PVR).

**Methods:**

In a prospective descriptive-analytic study, 146 female candidates for UDS from January 2016 to June 2017 entered the study. Patients were examined for urinary tract infection before UDS (up to 5 days before USD) and were enrolled in the study if they did not have bacteriuria or urinary tract infection. Patients did not receive antibiotic prophylaxis before performing UDS. The patients were asked to do U/A and U/C three days after the UDS test.

**Results:**

Among the 146 patients, 9 (6.2%) patients had considerable bacteriuria and 7 (4.8%) patients had UTI. The mean maximum detrusor pressure during urination and abnormal PVR before UDS had a significant correlation with positive urinary cultures after UDS (p<0.05).

**Conclusion:**

The results showed that this diagnostic procedure is low risk and the prophylactic antibiotic therapy is not required before UDS in pelvic floor clinic. It seems that prophylactic antibiotic therapy is only appropriate in case of PVR greater than 50 ml and possibly of the high detrusor pressure.

Urodynamic study is a diagnostic method in the field of pelvic floor disorders. This test requires catheterization of the urethra to inject the fluid for measurement of the bladder and urethral pressure. One of the complications of urethral catheterization is urinary tract infection due to damaging the epithelium of the urinary tract and development of suitable conditions for bacterial growth. It has been seen that urinary catheterization is associated with 1 to 5% chance of urinary tract infection per insertion (1). Different rates of urinary tract infections following UDS have been reported (1.5 to 30%) (2, 3) which can be attributed to factors such as the difference in study populations in terms of age or underlying problem, UDS performance method, and different definitions of urinary tract infections. There is no agreement on using prophylactic antibiotics before the UDS (4, 5). Old age patients (5), a low average of urine flow rate less than 7 mL/second (6) or a history of UTI within the past 4 weeks prior to the UDS are the high- risk factors for urinary tract infections (7). The aim of this study was to determine the rate of UTI after UDS in patients referred to the pelvic floor clinic with regard to their specific conditions, such as having pelvic organ prolapse and high post-voiding residual volume (PVR).

## Methods

**Study design and target group: **In a cross-sectional descriptive-analytic study, 156 female candidates for UDS from January 2016 to June 2017 entered the study. All female candidates for UDS who referred to the pelvic floor clinic and completed the inform consent were included in this study while presence of bacteriuria before the UDS, receiving antibiotic for any reason, having a fixed urine catheter, routine use of clean intermittent catheterization (CIC), urinary tract anomaly, recurrent UTI were the exclusion criteria in this study.

Patients were checked for urinary tract infection before UDS (up to 5 days before UDS) and were enrolled in the study if they did not have bacteriuria or urinary tract infection. A complete history of obstetric events, menopause status, previous surgery, frequent urinary tract infection, urinary stones, underlying diseases such as diabetes and thyroid diseases were recorded for the patients. The quantity of pelvic organ prolapse was determined by POP-Q system. Patients did not receive prophylactic antibiotic before performing UDS, and UDS tests were done routinely by the same nurse. The patients were asked to do clean midstream U/A and U/C three days after the UDS test and submit the result to our center by telephone, internet, or in the next visit. All patients are advised to contact us if they have symptoms of a urinary tract infection (dysuria, frequency, urinary urgency or supra pubic pain).Urinary tract infections (UTI) was defined in symptomatic subjects with a urine culture containing >100,000 CFU/mL (8). We defined “considerable bacteriuria” as > 10^2 ^colony count in U/C. The primary outcomes include the bacteriuria, UTI, and post voiding residual volume (PVR). Urinary symptoms are the secondary outcome.

**Data analysis: **Data were analyzed using SPSS Version 23 (SPSS Inc., Chicago, IL, USA). ANOVA was used for analysis of quantitative variables between groups.

## Results

 Of the 156 patients enrolled in the study, 10 patients were excluded from the study due to lack of follow up and not performing urinary culture after UDS and finally, 146 patients completed the study. The mean age of participants was 52.5 years and the mean BMI was 28.8 kg/m^2^ (Table 1).

One-way ANOVA showed that the mean maximum pressure of the detrusor during urination in the patients with considerable bacteriuria was significantly higher compared with those with negative culture and it was higher in patients with UTI compared with in patients with the considerable bacteriuria (P<0.05) (table 1). 

Urinary tract infection had no significant relationship with other results of UDS (P>0.05). A total of 82 (56.2%) patients had urinary leakage. Valsalva leak point pressure description is presented in table 2. As indicated in table 2, 9)6.2%) patients had considerable bacteriuria and 7(4.8%) suffered from UTI. Individuals with considerable bacteriuria were asymptomatic. Five patients with >10^5^colony count in U/C suffered from dysuria and frequency and two patients had hematuria. All patients with urinary tract infection received antibiotic.

**Table 1:** The mean of different variables of urodynamic according to the result of urine culture in the patient

**P- value**	**>=10** ^5^ **CFU/mL**		**10** ^2^ **-10** ^4^ **CFU/mL**		**No growth**		**variable**
	standard deviation	mean	standard deviation	mean	standard deviation	mean	
0.49	13.9	53.9	13.7	56.9	10.3	52.5	age
0.57	2.3	26.4	4.8	27.7	5.6	28.9	BMI
0.65	4.04	20.3	3.5	19.7	11.6	19.7	maximum flow rate before UDS
0.68	4.04	7.7	3.8	6.9	4.1	7.8	Average flow rate before UDS
0.50	52.5	101	52.9	135.8	52.7	116.3	Volume at first desire
0.58	84.8	253.4	142.9	243	80.9	224.2	Volume at a normal desire
0.72	73.5	409.9	174.6	434.4	120.1	389.9	Volume at strong desire
0.87	9.1	17.1	7	20.3	13.7	19.8	Maximum flow rate after UDS
0.02	55.6	68.4	23.4	57.2	23.6	42.6	Detrusor pressure at the maximum flow rate
0.62	3.2	6.4	2.3	8.3	3.7	7.6	Average flow rate after UDS

Among patients with negative culture, 20 patients suffered from dysuria and frequency, which were spontaneously recovered with fluid intake (table 2). Chi-square test showed that there was a significant correlation between the abnormal first PVR (pre-test) and urinary cultures (p<0.05). Patients with first PVR above 50 ml had considerable bacteriuria or UTI after UDS .Also, there was a significant relationship between diabetes and urine culture (p<0.05) (table 3). 

No significant correlation was found between the results of urine culture and other variables such as menopause, urine leak (p>0.05).

**Table 2:** Description of Valsalva leak point pressure and urine culture results

**Percentage**	**Number**	**Level**	**Variables**
43.8	64	non	Valsalva leak point pressure (CmH_2_O)
14.4	21	<60	
19.2	28	60-90	
22.6	33	>90	
89	130	No growth	Urine culture (CFU/ml)
6.2	9	10^2^-10^5^	
4.8	7	>=10^5^	

**Table 3:** Frequency of different cases via the outcome of urine culture

**p-value**	**>=10** ^5^ ** CFU/mL**		**Considerable bacteriuria**		**No growth**		**variable**	
	%	N	%	N	%	N		
0.001	4.4	6	4.4	6	91.2	124	<50cc	PVR before UDS
	33.3	1	66.7	2	0	0	>50cc	
0.65	4.2	4	7.3	7	88.5	85	<50cc	PVR after UDS
	6	3	4	2	90	45	>50cc	
0.91	5.3	3	5.3	3	89.4	51	no	Menopause
	4.6	4	6.9	6	88.5	77	yes	
0.03	5.6	7	4	5	90.4	114	no	Diabetes
	0	0	20	4	80	16	yes	

## Discussion

Among the 146 patients in this study, 7 patients had urinary tract infection (>=10^5^ CFU/mL) after UDS. Nine patients had considerable bacteriuria that was asymptomatic. On the other hand, there were 20 symptomatic patients who were culture-negative. These patients became asymptomatic after receiving enough fluids. Due to the low frequency of urinary tract infection after UDS, it seems that this diagnostic procedure is low risk and screening for urinary tract infection is not required after UDS; also, prophylactic antibiotic therapy is not recommended. A Review study in 2011 suggested that Prophylactic antibiotics did reduce the risk of bacteriuria after urodynamic studies but there was not enough evidence to suggest that this effect reduced symptomatic urinary tract infections (9). In our study all patients with first PVR above 50 ml had considerable bacteriuria or UTI after UDS. In Antibiotic prophylaxis in urodynamic guideline post-void residual volume > 100ml is a risk factor for tract urinary infection and with a one risk factor antibiotic prophylaxis is optional (10). In P-Quek et al. study, it was stated patients with high PVR had a higher chance of urinary tract infection, though the difference was not statistically significant which can be attributed to small sample size (11). In this study, there was no correlation between abnormal PVR (>50 ml) after UDS and urinary tract infection. PVR before and after UDS showed a statistically significant difference in our study (4.5 ml versus 67.2 ml), although this increase in PVR after UDS was expected due to presence of catheters and manipulations. This study showed that UTI was 7.3% (6 of 82) prevalent in patients with diagnosis of SUI in UDS, while it was 1.6% (1 of 64) prevalent among patients without urinary leakage. Although these differences were not statistically significant, the results have differed and further studies are required.

In this study, there was a significant relationship between high-pressure detrusor during urinary drainage and UTI. The importance of urethral obstruction in the development of urinary tract infections after UDS is also reported in the Quek study (11). In Shih-Wei Tsai et al’s. study, decreased average flow rate was reported as a risk factor for UTI after UDS (12) but this difference was not seen in the current study. There was a significant relationship between diabetes and considerable bacteriuria after UDS. The catheterization of the urethra can be responsible for increasing the considerable bacteriuria in diabetic patients. There was no infection in diabetic group and we think it was because of a small sample size. More than 3 normal vaginal delivery, presence of UTI before UDS, diabetes, and low average flow rate (<7 ml/second) are the risk factors for UTI after UDS and patients with this condition should take prophylactic antibiotics after UDS (12).

Pannek J suggested that antibiotic prophylaxis should be given because of a high rate of UTI following UDS (13), while Böthig R et al. concluded that under certain conditions such as bacteriuria, and reflex voiding before UDS, prophylactic antibiotic is required (14). Lathe et al. suggested in a meta-analysis that for the prevention of UTI after UDS we ought to give prophylactic antibiotics to 13 patients (15). The Society of Urodynamics, Female Pelvic Medicine, and Urogenital Reconstruction (SUFU) in 2017 announced that all patients should be screened for symptoms of UTI and undergo dipstick urinalysis before UDS, if the clinician suspects UTI; the UDS should be postponed until UTI has been treated (16). However, further studies are needed to clarify this inconsistency. The low sample size and uncertainty of the standard sampling method are the limitations of this study. A systematic review (Cochrane) Showed that antibiotic prophylaxis decreases the rate of bacteriuria but does not reduce the rate of urinary tract infection after UDS, and also recommend that if the urinary tract infection after UDS is above 10%, the prophylactic antibiotic is recommended (1). So, different factors that might influence the risk of UTI need future confirmation via randomized trials. 

In conclusion** t**he rate of urinary tract infection after UDS in our pelvic floor clinic was about 4.8%. Thus, prophylactic antibiotic therapy seems to be unnecessary. It seems that antibiotic prophylaxis is appropriate only in cases with PVR above 50 ml before performing UDS and possibly the high-detrusor pressure.

## References

[1] Foon R, Tooz-Hobson P, Latthe P (2012). Prophylactic antibiotics to reduce the risk of urinary tract infections after urodynamic studies. Cochrane Database Syst Rev.

[2] Ku JH, Kim SW, Kim HH (2004). Patient experience with a Urodynamic study: a prospective study in 208 patients. J Urol.

[3] Gürbüz C, Güner B, Atis G, Canat L, Caskurlu T (2013). Are prophylactic antibiotics necessary for urodynamic study?. Kaohsiung J Med Sci.

[4] Mirone V, Franco M (2014). Clinical aspects of antimicrobial prophylaxis for invasive urological procedures. J Chemother.

[5] Dass AK, Lo TS, Khanuengkitkong S, Tan YL (2013). Bacteriuria and safety of female urodynamic studies. Int Urogynecol J.

[6] Tsai SW, Kung FT, Chuang FC (2013). Evaluation of the relationship between urodynamic examination and urinary tract infection based on urinalysis results. Taiwan J Obstet Gynecol.

[7] Hwang SI, Lee BS, Han ZA (2016). Factors related to the occurrence of urinary tract infection following a urodynamic study in patients with spinal cord injury. Ann Rehabil Med.

[8] Nicolle LE (2008). Uncomplicated urinary tract infection in adults including uncomplicated pyelonephritis. Urol Clin North Am.

[9] Zeng S, Zhang Z, Bai Y (2019). Antimicrobial agents for preventing urinary tract infections in adults undergoing cystoscopy. Cochrane Database Syst Rev.

[10] Egrot C, Dinh A, Amarenco G (2018). Antibiotic prophylaxis in urodynamics: Clinical practice guidelines using a formal consensus method. Prog Urol.

[11] Quek P, Tay LH (2004). Morbidity and significant bacteriuria after urodynamic studies. Ann Acad Med Singapore.

[12] Tsai SW, Kung FT, Chuang FC (2013). Evaluation of the Relationship between urodynamic examination and urinary tract infection based on urinalysis results. Taiwan J Obstet Gynecol.

[13] Pannek J, Nehiba M (2007). Morbidity of urodynamic testing in patients with spinal cord injury: is antibiotic prophylaxis necessary?. Spinal Cord.

[14] Böthig R, Fiebag K, Thietje R, Faschingbauer M, Hirschfeld S (2013). Morbidity of urinary tract infection after urodynamic examination of hospitalized SCI patients: the impact of bladder management. Spinal Cord.

[15] Latthe PM, Foon R, Toozs-Hobson P (2008). Prophylactic antibiotics in urodynamics: a systematic review of effectiveness and safety. Neurourol Urodyn.

[16] Cameron AP, Campeau L, Brucker BM (2017). Best practice policy statement on urodynamic antibiotic prophylaxis in the non-index patient. Neurourol Urodyn.

